# Molecular epidemiological study of Scrub Typhus in residence, farm and forest habitats from Yunnan Province, China

**DOI:** 10.1371/journal.pone.0301841

**Published:** 2024-04-16

**Authors:** Jia-Wei Tian, Yi-Chen Kong, Pei-Yu Han, Fen-Hui Xu, Wei-Hong Yang, Yun-Zhi Zhang

**Affiliations:** 1 Yunnan Key Laboratory of Screening and Research on Anti-pathogenic Plant Resources from Western Yunnan, Yunnan Key Laboratory of Zoonotic Disease Cross-border Prevention and Quarantine, Institute of Preventive Medicine, School of Public Health, Dali University, Dali, Yunnan, China; 2 Yunnan Institute of Endemic Diseases Control and Prevention, Dali, Yunnan, China; Huadong Research Institute for Medicine and Biotechniques, CHINA

## Abstract

The number of people suffering from scrub typhus, which is not of concern, is increasing year by year, especially in Yunnan Province, China. From June 1, 2021 to August 15, 2022, a total of 505 mammalian samples were collected from farm, forest, and residential habitats with high incidence of scrub typhus in Yunnan, China, for nPCR (nested PCR) and qPCR (quantitative real-time PCR) detection of *Orientia tsutsugamushi*. A total of 4 orders of murine-like animals, Rodentia (87.52%, n = 442), Insectivora (10.29%, n = 52), Lagomorpha (1.79%, n = 9) and Scandentia (0.40%, n = 2) were trapped. Comparing the qPCR infection rates in the three habitats, it was no significant difference that the infection rate of residential habitat (44.44%) and that of the farm habitat (45.05%, P>0.05), which is much larger than that of the forest habitat (3.08%) (P<0.001). Three genotypes (Karp-like, Kato-like and TA763-like) of *O*. *tsutsugamushi* were found from Yunnan, China in this study.

## Introduction

As a re-emerging unclassified acute febrile infectious disease, scrub typhus, caused by *Orientia tsutsugamushi*, has a serious impact on the public health of Asia-Pacific countries. The World Health Organization has declared scrub typhus one of the most underdiagnosed/underreported diseases in the world. It has been stated two decades ago that more than 1 million cases of scrub typhus occur each year and 1 billion people are at risk of disease exposure in an habitat of more than 13 million square kilometers endemic for scrub typhus [1]. In southern China, the predicted high-risk habitats for scrub typhus transmission are mainly concentrated in the five Province of Yunnan, Guangxi, Guangdong, Hainan and Fujian, and it is estimated that more than 162 million people live in potential infection risk habitats in southern China [2]. From 2006 to 2016, the total number of reported and treated cases of scrub typhus in China increased from 254 to 21,562, and the annual incidence rate also increased dramatically, from 0.09/100,000 to 1.60/100,000 population [3]. In Yunnan province, which accounts for nearly a quarter of all scrub typhus infections in China, clinical diagnosis mostly relies on field activity history, as sensitive laboratory diagnosis of scrub typhus is difficult in resource-limited habitats, especially in border regions [4]. It is believed that a better understanding of the epidemic situation will help guide policymakers to formulate effective regional control strategies. This study conducted a host risk assessment of scrub typhus to determine the topography and direction of disease transmission in Yunnan Province, China.

The genus *Orientia* belongs to the order Rickettsiales within the family Rickettsiaceae. Chigger mites are currently the only known vector of scrub typhus. They have seven basic stages: the egg, deutovum (prelarva), larva, nymphochrysalis, nymph, imagochrysalis and adult.The larva (chiggers) is the only ectoparasitic stage [5]. However, *O*. *tsutsugamushi* is vectored by the biting of the larval life stage of infected chigger mites (e.g. *Leptotrombidium deliense*, *L*.*scutellare*) [6]. The species diversity of chigger mites in Yunnan was much higher than diversities reported previously in the other Provinces of China and in other countries. Small mammals (e.g. rodents and insectivores) are the most common hosts of chiggers [7]. They play an important role in the transmission of scrub typhus in nature as they can transport scrub typhus-carrying chiggers from endemic to non-endemic habitats. Scrub typhus is often transmitted to humans through the bite of chigger mites [8]. Therefore, the impact of scrub typhus on humans can be judged and the risk estimated by studying the infection rate of small mammals.

## Materials and methods

### Ethics statement

The number of scrub typhus cases was retrieved from the Yunnan Institute of Endemic Diseases Control and Prevention and no personally identifiable information was used as part of this study. This research was approved by the Medical Ethics Committee of Dali University under number DLDXLL2018008. All animals were treated according to the Guidelines of Regulations for the Administration of Laboratory Animals (Decree No. 2 of the State Science and Technology Commission of the People’s Republic of China, 1988) and the Guidelines for Treating Animals Kindly from Ministry of Science and Technology of the People’s Republic of China. All efforts were made to minimize discomfort to the animals.

### Data collection and sampling

We obtained the number of scrub typhus cases in Yunnan Province from 2006 to 2022 through the Yunnan Institute of Endemic Diseases Control and Prevention, and we obtained population data for calculating incidence from the National Bureau of Statistics of China (http://data.stats.gov.cnenglish/index.htm). We calculated annual incidence using the number of human scrub typhus cases divided by the corresponding population number at the end of a given year.

**Fig 1:**
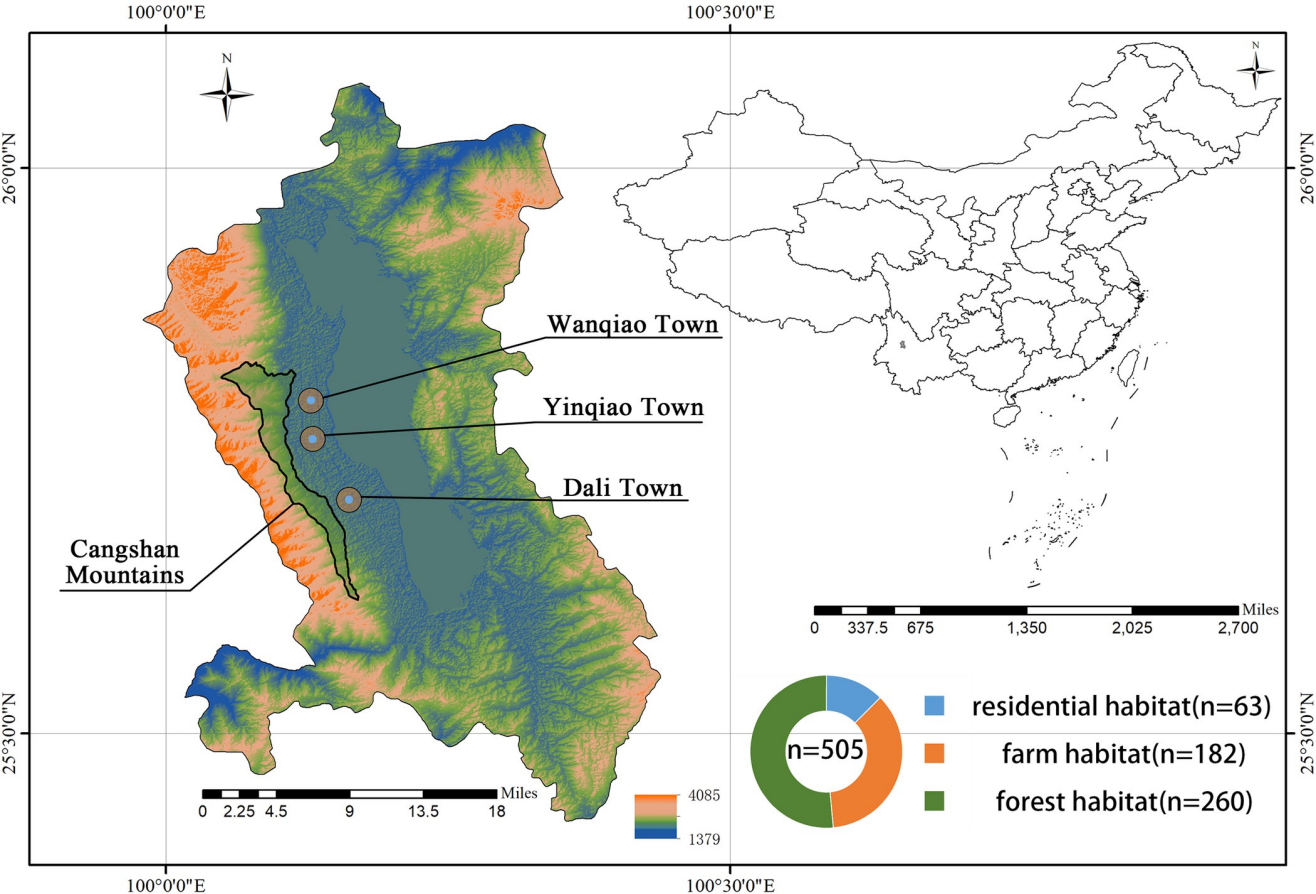
*Orientia tsutsugamushi* in small mammals, Yunnan Province, China, June 2021-August 2022. The upper right figure is a map of China, the lower right figure is a sector diagram showing the sampling volume of each habitat, the left figure is a map of Dali city, the brown area represent the sampling area of farm habitat; the blue area represent the sampling area of residential habitat; the green area represent the sampling area of forest habitat. The map was prepared in ArcGIS 10.8 using political boundaries from the National Geomatics Center of China (http://www.ngcc.cn/ngcc) for illustrative purposes only, these data are available free of charge.

Using fried food as bait and placing mouse live traps (20×12×10.5 cm Xiangyun Hong Jin Mouse Cage factory, Dali, Yunnan, China), the small mammal investigation was carried out from farm habitats (areas of crop and vegetable cultivation and production activities), forest habitats (areas where woody plants grow predominantly include coniferous forests, broadleaf forests, and mixed coniferous-broadleaf forests) and residential habitats (people living and residing with livestock) in Dali City, Yunnan Province, China from June 1, 2021 to August 15, 2022 (Fig 1). The small mammal were captured with 200 cages every day which were generally set every five meters and were arranged in the evening and collected in the morning, for approximately 6 days every habitat. The collected animal samples were immediately brought back to the laboratory for morphological identification [9]. Murine-like animals were anesthetized in an induction chamber containing cotton embedded with isoflurane (1 ml of isoflurane per 500 ml of chamber volume) [10]. After anaesthesia, the murine-like animals was sacrificed on a warming blanket to minimize distress. The sacrificed mammals were dissected to collect the heart, liver, spleen, lung, kidney, and intestinal tissues placed in 2 mL cryogenic vials (CORNING, Shanghai, China) which were stored at -80°C for further analysis. The mitochondrial cytochrome b (mt-Cytb) gene of liver tissue DNA was amplified by PCR (Bio-Gener, Hangzhou, China) for molecular biological identification [11]. The species, geographical location, altitude(Beidou Navigation Satellite System, China) and air humidity (Peakmeter, Shenzhen, China) of the captured animals were recorded.

### Nucleic acid extraction

The collected mammalian spleen tissue for detection were aseptically clipped, and about 0.1 g of spleen tissue was placed into the GeneReady animal PIII pulverization tube (Life Real, Hangzhou, China) to which sterilized 500 μL of phosphate-buffered saline (PBS) with a pH of 7.4 had been added. Grinding was performed in a GeneReady Ultimate biological sample cryogenic rapid preparation centrifuge system (Life Real, Hangzhou, China). Nucleic acid extraction from animal tissue grinding fluid (200μL) was performed using a DNA extraction kit (TIANGEN, Beijing, China) according to the manufacturer’s instructions.

#### Conventional PCR

The genotype of *O*. *tsutsugamushi* in host animals was determined by nested polymerase chain reaction (nPCR) using the 56 kDa outer membrane protein gene [12]. Two sets of primers used were as follows: outer primers, Ot-Out-F1 (5’-TACATTAGCTGCGGGTATGACA-3’), Ot-OutR1 (5’-CCAGCATAATTCTTCAACCAAG-3’) and inner primers Ot-In-F2 (5’-GAGCAGAGCTAGGTGTTATGTA-3’), Ot-In-R2 (5’-TAGGCATTATAGTAGGCTGAGG-3’).

#### Standardisation of qPCR

According to the method we previously described [13], it was amplified that the 47 kDa gene of 1401 bp of the *O*. *tsutsugamushi* Karp strain, and were cloned into the pEASY-T1 vector (TransGen Biotech, Beijing, China), and the T-loaded products were transformed into DH5α *E*. *coli* cells. *E*. *coli* cells containing target DNA on LB medium were grown in LB medium for 9 hours. Plasmid DNA was extracted from 600μl of the suspension, using the Plasmid Mini Kit I (Omega Bio-Tek, Norcross, GA, America), following the manufacturer’s instructions and the concentration was determined. The *O*. *tsutsugamushi* data in ng/μl were then converted to numbers of copies/μl. For plasmid detection sensitivity, we performed real-time quantitative polymerase chain reaction (qPCR) with serial dilutions of plasmid DNA from 5×10^9^ copies/μl to 5×10^3^ copies/μl using DNase-free deionized water to create a standard curve.

The presence of *O*. *tsutsugamushi* was assessed by qPCR and the degree of infection in the host animals was determined using the 47 kDa high temperature transmembrane protein gene. qPCR was accomplished using probe (FAM-TGGGTAGCTTTGGTGGACCGATGTTTA ATCT-BHQ1) to determine *O*. *tsutsugamushi* copy numbers [14]. All qPCRs were run in triplicate in Applied Biosystems’ QuantStudio 3 (Thermo Fischer Scientific, Waltham, MA, USA).

#### Phylogenetic and data analysis

All data were statistically analyzed using SPSS 24.0 statistical software. The Chi-square test was used for statistical methods, and P <0.05 was considered statistically significant.

The measured sequence was compared with the nucleotide sequence of *O*. *tsutsugamushi* in GenBank by BLAST algorithm and the nucleotide and amino acid sequences of similar strains were obtained. The sequence was compared with 7 similar strains using the BioAider program [15] (Additional file: S1 Table), and then the phylogenetic tree was constructed using the neighbor-joining method (NJ method) in the MEGA Ⅹ software [16]. The bootstrap value was 1000, and the distance was determined by the maximum likelihood method.

## Results

**Fig 2:**
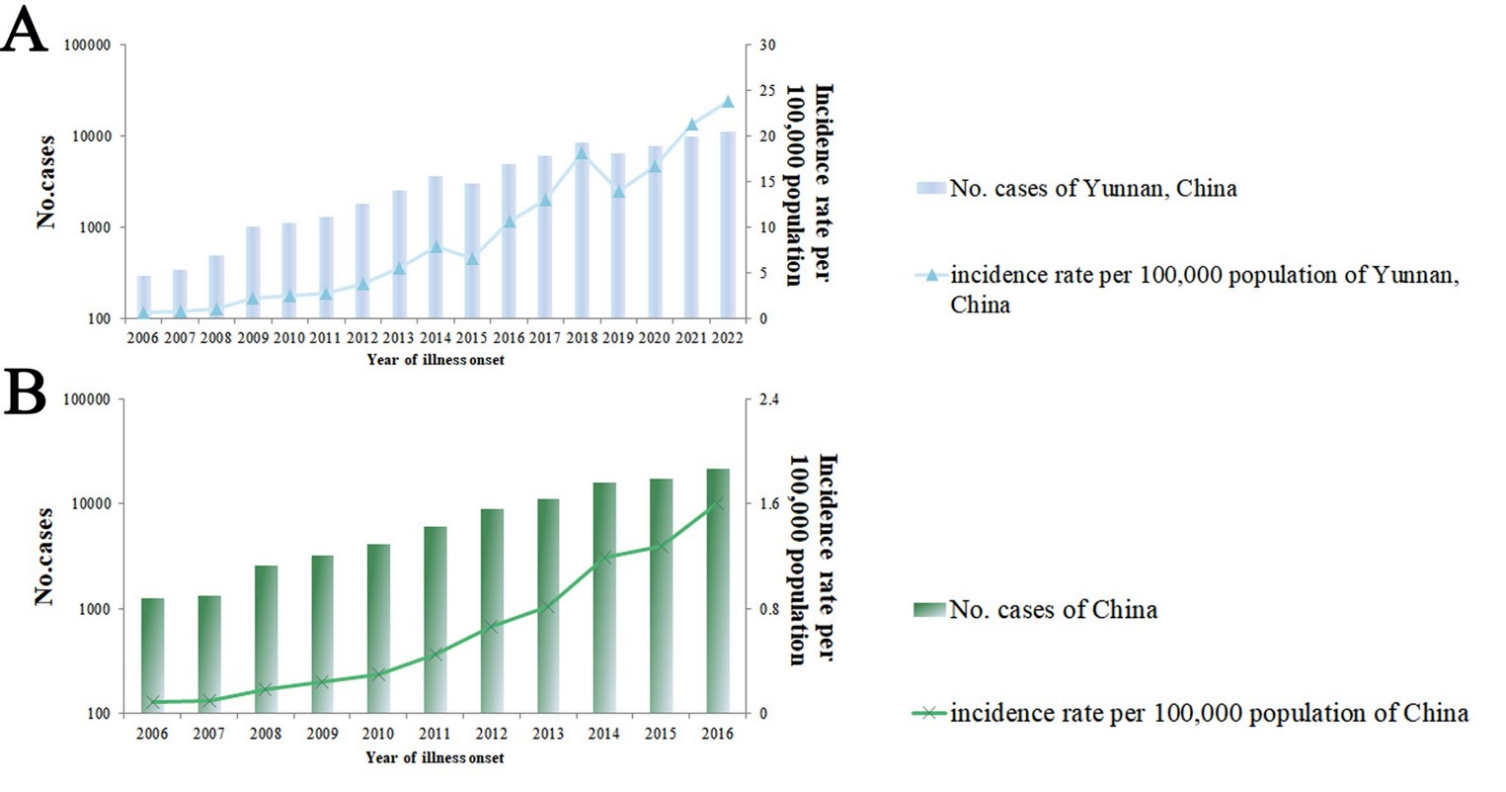
Reported cases of scrub typhus. (A) Cases in Yunnan, China, 2006–2022. (B) Cases in China, 2006–2016.

The total number of scrub typhus cases in Yunnan Province increased from 292 to 11,189, and the annual incidence rate increased from 0.65/100,000 to 23.84/100,000 population from 2006 to 2022. As of December 2022, compared to the end of 2019, the incidence rate of infections had grown by 71.14% from 13.93/100,000 to 23.84/100,000 population (Fig 2).

The mammal samples were collected from high-incidence habitats of scrub typhus in Yunnan Province, China from June 1, 2021 to August 15, 2022. A total of 505 mammal samples were collected from farm habitat (36.04%, n = 182), forest habitat (51.49%, n = 260) and residential habitat (12.47%, n = 63).

We captured a total of 505 small mammals belonging to 20 species in 4 orders. The 4 orders of murine-like animal are Rodentia (87.52%, n = 442), Insectivora (10.29%, n = 52), Lagomorpha (1.79%, n = 9) and Scandentia (0.40%, n = 2). The dominant species in the farm, residential and forest habitats are the Chevrieri’s field mouse (*Apodemus chevrieri*), Asian house rat (*Rattus tanezumi*) and *A*. *chevrieri*, respectively. High infection rates in murine-like animals were observed in qPCR results in residential and farm habitats. Comparing the qPCR infection rates in the three habitats, it was no significant difference that the infection rate of residential habitat (44.44%) and that of the farm habitat (45.05%) (P>0.05), but the infection rate in the residential habitat and the farm habitat are much larger than that of the forest habitat (3.08%) (P<0.001). The total positive rate of qPCR was 23.56%.

The nPCR infection rate in the residential habitat was consistent with that in the farm habitat, both of which were higher than those in the forest habitat (P<0.001) (Table 1). The nucleotide and amino acid sequences of the 56 kDa gene sequences obtained in this study were compared with similar strains (Additional file: S2 Table).

**Table 1:** Prevalence of *Orientia tsutsugamushi* in small mammals, Yunnan Province, China, June 2021-August 2022.

Topography	Order	Species	Number of detections	qPCR positive rate	qPCR DNA copies/μl	nPCR positive rate
Farm habitat	Rodentia	*Apodemus chevrieri*	112	51/112 (45.54%)	55.76	3/112 (2.68%)
		*Eothenomys cachinus*	34	14/34 (41.18%)	142.91	0
		*Rattus tanezumi*	14	6/14 (42.86%)	50.21	0
		*Rattus norvegicus*	4	0	-	0
		*Mus caroli*	3	3/3 (100%)	31.48	0
		*Mus musculus*	2	0	-	0
		*Niviventer fulvescens*	1	0	-	0
		*Rattus nitidus*	1	1/1 (100%)	357.43	0
		*Eothenomys miletus*	1	0	-	0
	Insectivora	*Crocidura attenuata*	8	6/8 (75%)	12.19	0
		*Suncus murinus*	2	1/2 (50%)	41.15	0
	Total		182	82/182 (45.05%)	58.02	3/182 (1.65%)
Forest habitat	Rodentia	*Apodemus chevrieri*	91	4/91 (4.40%)	17.77	0
		*Eothenomys cachinus*	81	3/81 (3.70%)	102.29	1/81 (1.23%)
		*Rattus tanezumi*	31	0	-	0
		*Niviventer eha*	3	0	-	0
		*Niviventer niviventer*	3	0	-	0
		*Niviventer fulvescens*	1	0	-	0
	Insectivora	*Anourosorex squamipes*	21	0	-	0
		*Crocidura attenuata*	11	1/11 (9.09%)	369.74	0
		*Episoriculus caudatus*	5	0	-	0
		*Neohylomys hainanensis*	3	1/3 (33.33%)	13.97	0
		*Scaptonyx fusicaudus*	1	0	-	0
	Lagomorpha	*Ochotona thibetana*	8	0	-	0
		*Ochotona roylii*	1	0	-	0
	Total		260	9/260 (3.08%)	43.44	1/260 (0.38%)
Residential habitat	Rodentia	*Rattus tanezumi*	36	16/36 (43.24%)	49.62	1/36 (2.78%)
		*Rattus norvegicus*	23	9/23 (39.13%)	36.56	2/23 (8.70%)
		*Eothenomys cachinus*	1	1/1 (100%)	72.62	1/1 (100%)
	Scandentia	*Tupaia belangeri*	2	2/2 (100%)	17.39	0
	Insectivora	*Suncus murinus*	1	0	-	0
	Total		63	28/63 (44.44%)	42.19	4/63 (6.35%)
Total			505	119/505 (23.56%)	52.47	8/505 (1.58%)

**Fig 3:**
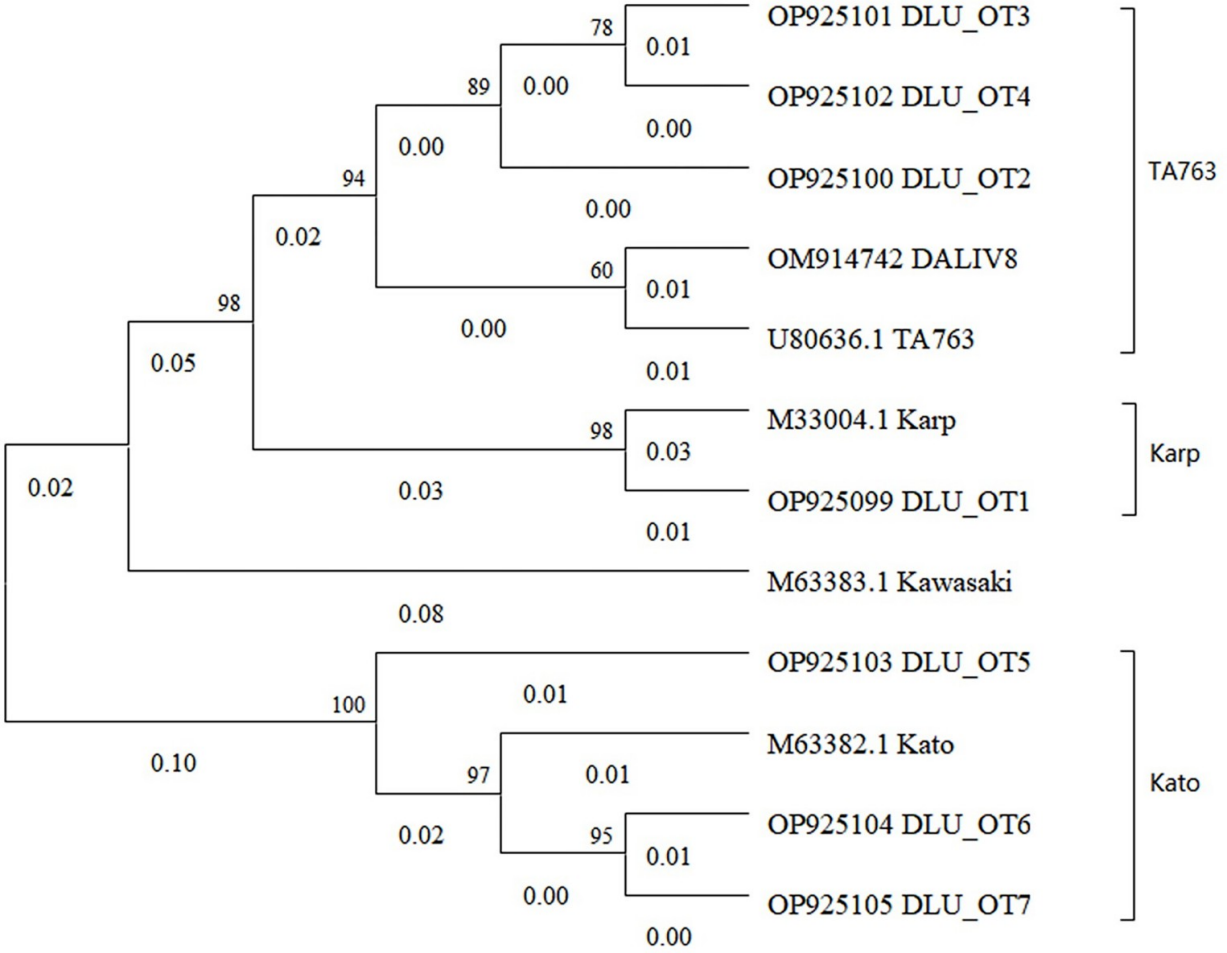
Phylogenetic trees of partial 56 kDa gene sequences of *O*. *tsutsugamushi* at the nucleotide level.

Phylogenetic analysis showed that the 3 genotypes from 8 strains of 56 kDa gene sequences were obtained, including 3 sequences (Accession no: OP925103-OP925105) clustered with Kato genotype, 4 sequences (Accession no: OM914742, OP925100-OP925102) clustered with TA763 genotype, and 1 sequence (Accession no: OP925009) clustered with Karp genotype (Fig 3). The Karp-like genotype *O*. *tsutsugamushi* detected in this experiment had 98.8% nucleotide homology with a Yunnan patient in the NCBI (Accession no: KY971308). The three detected strains of TA763-like were 98.8%-99.4% with the patient (Accession no: MW495582) and chigger (Accession no: GU120142) from Taiwan in the NCBI. In addition, there are three strains of Kato genotype *O*. *tsutsugamushi*, which have 98.65%-99.32% nucleotide homology with Yunnan patient (Accession no: KY971312) and Thailand patient (Accession no: OP548067) in the NCBI (https://www.ncbi.nlm.nih.gov), respectively.

## Discussion

This study collected the latest scrub typhus case data, and found that both the number of scrub typhus cases and the incidence rate are still on the rise by the end of 2022 in Yunnan, China. This requires us to identify the potential relationship between scrub typhus and its related factors as soon as possible, so as to curb the rapid spread of this disease, which poses a major threat to human health. Therefore, we collected murine-like animal samples to explore the relationship between scrub typhus and human habitats.

Among the 505 samples in this experiment, three genotypes were found to reflect the diversity of *O*. *tsutsugamushi* genotypes in Yunnan, China. The TA763 genotype was first isolated from *Rattus rajah* in Thailand [17]. The TA763-like sequence obtained in this study is the most similar to the sequence obtained in Taiwan, which can be considered that *O*. *tsutsugamushi* may have migrated over long distances. Additionally, the 56 kDa gene of the three Kato-like genotypes obtained in this study was most similar to that of patients in Yunnan and Thailand, respectively. However, researchers have found that the most common genotypes in Thailand are Karp and Karp-related [18, 19]. In the reports from China, the dominant genotypes of scrub typhus in Yunnan were Karp and Kato. Different genotypes of *O*. *tsutsugamushi* have a tendency to spread within “tsutsugamushi triangle” [20]. This result suggests that 56 kDa gene sequence detection plays a role in establishing cross-border emerging infectious disease linkages. In this experiment, the dominant genotypes of scrub typhus are considered as Kato-like and TA763-like in Yunnan Province during 2021–2022.

The terrain of Yunnan is dominated by plateaus [21]. This experiment found that the infection rate in forest habitats is lower than that in farm land and residential habitats. It may be considered that the forests in Southwest China are dominated by high altitudes, and the lower temperature in high altitude habitats is not suitable for the survival of chiggers [22, 23]. In addition, Yunnan Province is adjacent to several countries with high incidence of scrub typhus [24–26]. Therefore, it is necessary to study scrub typhus, an emerging infectious disease, because the incidence rate in Yunnan Province has been increasing year by year. In a previous study of hosts and parasites in Yunnan Province, the researchers collected 10,222 small mammals of 62 species that were infested by 92,990 chiggers of 224 species which can observe the species diversity of scrub typhus hosts and vectors in Yunnan Province [27]. In China, the burden of disease caused by neglected tropical diseases such as scrub typhus will not be underestimated. In animal hosts, emerging pathogens may not cause symptoms in host animals. However, while these pathogens are not always in the host animal, the pathogen evolves in small animals until it spills over into humans [28]. Chiggers are ectoparasites of small mammals, and chiggers infected with *O*. *tsutsugamushi* are transmitted to humans by biting. [29]. Our results indicate that the infection rate of *O*. *tsutsugamushi* among rodents in habitats with high human activity is higher than that of in habitats with low human activity, which may be one of the reasons why the incidence of Scrub typhus in Yunnan Province has been increasing year by year.

We found an increased risk of exposure to *O*. *tsutsugamushi* in residential habitats. The difference of infection rate of *O*. *tsutsugamushi* in hosts indicated that scrub typhus tended to move from forests to farm and into cities. In 2021, an ecological analysis of scrub typhus in Thailand found that, forest habitat was poorly associated with *O*. *tsutsugamushi* infection in all the analyses [23]. The infection rate of *O*. *tsutsugamushi* in murine-like animals was positively correlated with the disturbance level of human activities. This finding is inconsistent with the cognition of previous articles on the discovery of scrub typhus infection [30]. This may explain one reason of the increasing number of scrub typhus cases in Yunnan Province year by year [31]. The reason for this is speculated to be the reduction of forest habitat in Yunnan and the replacement of forests with farm or residential habitats [32]. The second reason is to suspect that the emergence of global warming is causing geographic migration of rodent hosts carrying scrub typhus to warmer, wetter locations [33]. The occurrence of both of these situations will have the result of the migration of rodents to residential habitats [34]. It is suggested that the risk of scrub typhus may change significantly under the rapid environmental changes in humans [35].

## Conclusions

The infection rate of *O*. *tsutsugamushi* in small mammal was positively correlated with the disturbance level of human activities, and this result is consistent with the increase in the number of infections in Yunnan over the same period. The results of this study suggesting that *O*. *tsutsugamushi* can be infected without outdoor activities, which is helpful for disease control and diagnostic strategies.

## Supporting information

S1 Table Information of the sequences obtained in this study and other reference strains. (DOCX)

S2 Table Nucleotide and amino sequence alignment results of 56kDa type-specific antigen gene of the sequences obtained in this study with other reference strains. (DOCX)

S1 File (DOCX)

## References

[1] Walker DH. Scrub Typhus—Scientific Neglect, Ever-Widening Impact. N Engl J Med. 2016;375:913–5. doi: 10.1056/NEJMp1608499 . 27602663

[2] Zheng C, Jiang D, Ding F, Fu J, Hao M. Spatiotemporal Patterns and Risk Factors for Scrub Typhus From 2007 to 2017 in Southern China. Clin Infect Dis. 2019;69:1205–11. doi: 10.1093/cid/ciy1050 . 30535175

[3] Li Z, Xin H, Sun J, Lai S, Zeng L, Zheng C, et al. Epidemiologic Changes of Scrub Typhus in China, 1952–2016. Emerg Infect Dis. 2020;26:1091–101. doi: 10.3201/eid2606.191168 . 32441637 PMC7258452

[4] Saraswati K, Day NPJ, Mukaka M, Blacksell SD. Scrub typhus point-of-care testing: A systematic review and meta-analysis. PLoS Negl Trop Dis. 2018;12:e0006330. doi: 10.1371/journal.pntd.0006330 . 29579046 PMC5892940

[5] Peng PY, Guo XG, Ren TG, Dong WG, Song WY. An updated distribution and hosts: trombiculid mites (Acari: Trombidiformes) associated with small mammals in Yunnan Province, southwest China. Parasitol Res. 2016;115:1923–38. doi: 10.1007/s00436-016-4934-4 . 26833324

[6] Mullen GR, Durden LA. Medical and veterinary entomology: Academic press; 2009.

[7] Alkathiry H, Al-Rofaai A, Ya’cob Z, Cutmore TS, Mohd-Azami SNI, Husin NA, et al. Habitat and Season Drive Chigger Mite Diversity and Abundance on Small Mammals in Peninsular Malaysia. Pathogens. 2022;11:1087. doi: 10.3390/pathogens11101087 . 36297144 PMC9607564

[8] Kuo CC, Lee PL, Chen CH, Wang HC. Surveillance of potential hosts and vectors of scrub typhus in Taiwan. Parasit Vectors. 2015;8:611. doi: 10.1186/s13071-015-1221-7 . 26626287 PMC4666075

[9] Zheng Z, Jiang Z, Chen A. Rodentology. 2nd ed. China: Shanghai Jiaotong University Press; 2012. [in Chinese].

[10] Acosta-Jamett G, Martínez-Valdebenito C, Beltrami E, Silva-de La Fuente MC, Jiang J, Richards AL, et al. Identification of trombiculid mites (Acari: Trombiculidae) on rodents from Chiloé Island and molecular evidence of infection with *Orientia* species. PLoS Negl Trop Dis. 2020;14:e0007619. doi: 10.1371/journal.pntd.0007619 . 31971956 PMC6999909

[11] Guillen-Servent A, Francis CM. A new species of bat of the Hipposideros bicolor group (Chiroptera: Hipposideridae) from Central Laos, with evidence of convergent evolution with Sundaic taxa. Acta Chiropt. 2006;8:39–61. 10.3161/1733-5329(2006)8[39:Ansobo]2.0.Co;2

[12] Furuya Y, Yoshida Y, Katayama T, Yamamoto S, Kawamura A Jr., Serotype-specific amplification of *Rickettsia tsutsugamushi* DNA by nested polymerase chain reaction. J Clin Microbiol. 1993;31:1637–40. doi: 10.1128/jcm.31.6.1637-1640.1993 . 8315007 PMC265595

[13] Xu FH, Han PY, Tian JW, Zong LD, Yin HM, Zhao JY, et al. Detection of Alpha- and Betacoronaviruses in Small Mammals in Western Yunnan Province, China. Viruses. 2023;15:1965. doi: 10.3390/v15091965 . 37766371 PMC10535241

[14] Jiang J, Chan TC, Temenak JJ, Dasch GA, Ching WM, Richards AL. Development of a quantitative real-time polymerase chain reaction assay specific for *Orientia tsutsugamushi*. Am J Trop Med Hyg. 2004;70:351–6. 10.4269/ajtmh.2004.70.351 . 15100446

[15] Zhou ZJ, Qiu Y, Pu Y, Huang X, Ge XY. BioAider: An efficient tool for viral genome analysis and its application in tracing SARS-CoV-2 transmission. Sustain Cities Soc. 2020;63:102466. doi: 10.1016/j.scs.2020.102466 . 32904401 PMC7455202

[16] Kumar S, Stecher G, Li M, Knyaz C, Tamura K. MEGA X: Molecular Evolutionary Genetics Analysis across Computing Platforms. Mol Biol Evol. 2018;35:1547–9. doi: 10.1093/molbev/msy096 . 29722887 PMC5967553

[17] Phuklia W, Panyanivong P, Sengdetka D, Sonthayanon P, Newton PN, Paris DH, et al. Novel high-throughput screening method using quantitative PCR to determine the antimicrobial susceptibility of *Orientia tsutsugamushi* clinical isolates. J Antimicrob Chemother. 2019;74:74–81. doi: 10.1093/jac/dky402 . 30295746 PMC6293087

[18] Blacksell SD, Luksameetanasan R, Kalambaheti T, Aukkanit N, Paris DH, McGready R, et al. Genetic typing of the 56-kDa type-specific antigen gene of contemporary *Orientia tsutsugamushi* isolates causing human scrub typhus at two sites in north-eastern and western Thailand. FEMS Immunol Med Microbiol. 2008;52:335–42. doi: 10.1111/j.1574-695X.2007.00375.x . 18312580

[19] Ruang-Areerate T, Jeamwattanalert P, Rodkvamtook W, Richards AL, Sunyakumthorn P, Gaywee J. Genotype diversity and distribution of *Orientia tsutsugamushi* causing scrub typhus in Thailand. J Clin Microbiol. 2011;49:2584–9. doi: 10.1128/JCM.00355-11 . 21593255 PMC3147819

[20] Richards AL, Jiang J. Scrub Typhus: Historic Perspective and Current Status of the Worldwide Presence of *Orientia* Species. Trop Med Infect Dis. 2020;5:49. doi: 10.3390/tropicalmed5020049 . 32244598 PMC7344502

[21] Han H, Liang Y, Song Z, He Z, Duan R, Chen Y, et al. Epidemiological Characteristics of Human and Animal Plague in Yunnan Province, China, 1950 to 2020. Microbiol Spectr. 2022;10:e0166222. doi: 10.1128/spectrum.01662-22 . 36219109 PMC9784778

[22] Steventon C, Harley D, Wicker L, Legione AR, Devlin JM, Hufschmid J. An assessment of ectoparasites across highland and lowland populations of Leadbeater’s possum (Gymnobelideus leadbeateri): Implications for genetic rescue translocations. Int J Parasitol Parasites Wildl. 2022;18:152–6. doi: 10.1016/j.ijppaw.2022.05.002 . 35586791 PMC9108725

[23] Elliott I, Thangnimitchok N, Chaisiri K, Wangrangsimakul T, Jaiboon P, Day NPJ, et al. *Orientia tsutsugamushi* dynamics in vectors and hosts: ecology and risk factors for foci of scrub typhus transmission in northern Thailand. Parasit Vectors. 2021;14:540. doi: 10.1186/s13071-021-05042-4 . 34663445 PMC8524837

[24] Elders PND, Dhawan S, Tanganuchitcharnchai A, Phommasone K, Chansamouth V, Day NPJ, et al. Diagnostic accuracy of an in-house Scrub Typhus enzyme linked immunoassay for the detection of IgM and IgG antibodies in Laos. PLoS Negl Trop Dis. 2020;14:e0008858. doi: 10.1371/journal.pntd.0008858 . 33284807 PMC7746293

[25] Elders PND, Swe MMM, Phyo AP, McLean ARD, Lin HN, Soe K, et al. Serological evidence indicates widespread distribution of rickettsioses in Myanmar. Int J Infect Dis. 2021;103:494–501. doi: 10.1016/j.ijid.2020.12.013 . 33310022 PMC7862081

[26] Trung NV, Hoi LT, Dien VM, Huong DT, Hoa TM, Lien VN, et al. Clinical Manifestations and Molecular Diagnosis of Scrub Typhus and Murine Typhus, Vietnam, 2015–2017. Emerging infectious diseases. 2019;25:633–41. doi: 10.3201/eid2504.180691 . 30882318 PMC6433017

[27] Zhan YZ, Guo XG, Speakman JR, Zuo XH, Wu D, Wang QH, et al. Abundances and host relationships of chigger mites in Yunnan Province, China. Med Vet Entomol. 2013;27:194–202. doi: 10.1111/j.1365-2915.2012.01053.x . 23167491

[28] Olival KJ, Hosseini PR, Zambrana-Torrelio C, Ross N, Bogich TL, Daszak P. Host and viral traits predict zoonotic spillover from mammals. Nature. 2017;546:646–50. doi: 10.1038/nature22975 . 28636590 PMC5570460

[29] Meerburg BG, Singleton GR, Kijlstra A. Rodent-borne diseases and their risks for public health. Crit Rev Microbiol. 2009;35:221–70. doi: 10.1080/10408410902989837 . 19548807

[30] Lin EC, Tu HP, Hong CH. Halved Incidence of Scrub Typhus after Travel Restrictions to Confine a Surge of COVID-19 in Taiwan. Pathogens. 2021;10:1386. doi: 10.3390/pathogens10111386 . 34832542 PMC8623167

[31] Peng PY, Xu L, Wang GX, He WY, Yan TL, Guo XG. Epidemiological characteristics and spatiotemporal patterns of scrub typhus in Yunnan Province from 2006 to 2017. Sci Rep. 2022;12:2985. doi: 10.1038/s41598-022-07082-x . 35194139 PMC8863789

[32] Shi L, Dossa GGO, Paudel E, Zang H, Xu J, Harrison RD. Changes in Fungal Communities across a Forest Disturbance Gradient. Appl Environ Microbiol. 2019;85:e00080–19. doi: 10.1128/AEM.00080-19 . 30979833 PMC6544830

[33] Wei Y, Huang Y, Li X, Ma Y, Tao X, Wu X, et al. Climate variability, animal reservoir and transmission of scrub typhus in Southern China. PLoS Negl Trop Dis. 2017;11:e0005447. doi: 10.1371/journal.pntd.0005447 . 28273079 PMC5358896

[34] Roberts T, Parker DM, Bulterys PL, Rattanavong S, Elliott I, Phommasone K, et al. A spatio-temporal analysis of scrub typhus and murine typhus in Laos; implications from changing landscapes and climate. PLoS Negl Trop Dis. 2021;15:e0009685. doi: 10.1371/journal.pntd.0009685 . 34432800 PMC8386877

[35] Ding F, Wang Q, Hao M, Maude RJ, John Day NP, Lai S, et al. Climate drives the spatiotemporal dynamics of scrub typhus in China. Glob Chang Biol. 2022;28:6618–28. doi: 10.1111/gcb.16395 . 36056457 PMC9825873

