# Supplementary material for: Molecular epidemiological study of Scrub Typhus in residence, farm and forest habitats from Yunnan Province, China
Source: PLoS One. 2024 Apr 16;19(4):e0301841. doi: 10.1371/journal.pone.0301841 (PMC11020965; doi:10.1371/journal.pone.0301841)
Supplement: S1 Table — (DOCX) [file pone.0301841.s001.docx]

**Table S1.** Information of the sequences obtained in this study and other reference strains.

| **No.** | **NCBI Accession no.** | **Strain name** | **Isolated location** | **Isolated country** | **Isolated year** | **Isolation host** |
| --- | --- | --- | --- | --- | --- | --- |
| 1 | U80636 | TA763 | Thailand | Thailand | 1963 | Rodent |
| 2 | M33004 | Karp | New Guinea | New Guinea | 1943 | Human |
| 3 | M63382 | Kato | Niigata, Japan | Japan | 1955 | Human |
| 4 | M63383 | Kawasaki | Miyazaki, Japan | Japan | 1981 | Human |
| 5 | KY971308 | YN16-25 | Yunnan, China | China | 2016 | Human |
| 6 | MW495582 | KL0807a | Taiwan, China | China | 2008 | Human |
| 7 | OP548067 | CREX029BU | Thailand | Thailand | 2019 | Human |
| 8 | KY971312 | YN16-52 | Yunnan, China | China | 2016 | Human |
| 9 | GU120142 | HL03-1 | Taiwan, China | China | 2008 | Chiggers |
| 10 | OM914742 | DALIV8 | Yunnan, China | China | 2015 | Rodent |
| 11 | OP925099 | DLU_OT1 | Yunnan, China | China | 2022 | Rodent |
| 12 | OP925100 | DLU_OT2 | Yunnan, China | China | 2022 | Rodent |
| 13 | OP925101 | DLU_OT3 | Yunnan, China | China | 2022 | Rodent |
| 14 | OP925102 | DLU_OT4 | Yunnan, China | China | 2021 | Rodent |
| 15 | OP925103 | DLU_OT5 | Yunnan, China | China | 2021 | Rodent |
| 16 | OP925104 | DLU_OT6 | Yunnan, China | China | 2021 | Rodent |
| 17 | OP925105 | DLU_OT7 | Yunnan, China | China | 2022 | Rodent |
