# Supplementary material for: Molecular epidemiological study of Scrub Typhus in residence, farm and forest habitats from Yunnan Province, China
Source: PLoS One. 2024 Apr 16;19(4):e0301841. doi: 10.1371/journal.pone.0301841 (PMC11020965; doi:10.1371/journal.pone.0301841)
Supplement: S2 Table — (DOCX) [file pone.0301841.s002.docx]

**Table S2.** Nucleotide and amino sequence alignment results of 56kDa type-specific antigen gene of the sequences obtained in this study with other reference strains.

|  | TA763 | Karp | Kato | Kawasaki | YN16-25 | KL0807a | CREX029BU | YN16-52 | HL03-1 | DALIV8 | DLU_OT1 | DLU_OT2 | DLU_OT3 | DLU_OT4 | DLU_OT5 | DLU_OT6 | DLU_OT7 |
| --- | --- | --- | --- | --- | --- | --- | --- | --- | --- | --- | --- | --- | --- | --- | --- | --- | --- |
| TA763 | - | 90.36 | 75.32 | 83.75 | 92.77 | 98.80 | 75.97 | 75.32 | 98.80 | 98.19 | 92.77 | 98.19 | 97.59 | 98.19 | 75.32 | 74.03 | 74.68 |
| Karp | 80.00 | - | 74.03 | 79.38 | 95.18 | 91.57 | 74.68 | 74.03 | 91.57 | 92.17 | 96.39 | 92.17 | 91.57 | 92.17 | 75.32 | 74.03 | 74.68 |
| Kato | 62.75 | 60.78 | - | 76.82 | 75.32 | 75.32 | 97.30 | 100.00 | 75.32 | 74.68 | 75.32 | 75.97 | 75.32 | 75.97 | 96.62 | 98.65 | 99.32 |
| Kawasaki | 75.47 | 64.15 | 64.00 | - | 80.62 | 83.75 | 78.15 | 76.82 | 83.75 | 83.12 | 80.62 | 83.12 | 82.50 | 83.12 | 77.48 | 75.50 | 76.16 |
| YN16-25 | 87.27 | 89.09 | 62.75 | 67.92 | - | 92.77 | 75.97 | 75.32 | 92.77 | 92.17 | 98.80 | 93.37 | 92.77 | 93.37 | 75.32 | 74.03 | 74.68 |
| KL0807a | 98.18 | 81.82 | 62.75 | 75.47 | 89.09 | - | 75.97 | 75.32 | 100.00 | 99.40 | 93.98 | 99.40 | 98.80 | 99.40 | 76.62 | 75.32 | 75.97 |
| CREX029BU | 62.75 | 60.78 | 95.92 | 64.00 | 62.75 | 62.75 | - | 97.30 | 75.97 | 75.32 | 75.97 | 76.62 | 75.97 | 76.62 | 99.32 | 95.95 | 96.62 |
| YN16-52 | 62.75 | 60.78 | 100.00 | 64.00 | 62.75 | 62.75 | 95.92 | - | 75.32 | 74.68 | 75.32 | 75.97 | 75.32 | 75.97 | 96.62 | 98.65 | 99.32 |
| HL03-1 | 98.18 | 81.82 | 62.75 | 75.47 | 89.09 | 100.00 | 62.75 | 62.75 | - | 99.40 | 93.98 | 99.40 | 98.80 | 99.40 | 76.62 | 75.32 | 75.97 |
| DALIV8 | 96.36 | 83.64 | 60.78 | 73.58 | 87.27 | 98.18 | 60.78 | 60.78 | 98.18 | - | 93.37 | 98.80 | 98.19 | 98.80 | 75.97 | 74.68 | 75.32 |
| DLU_OT1 | 87.27 | 89.09 | 62.75 | 67.92 | 100.00 | 89.09 | 62.75 | 62.75 | 89.09 | 87.27 | - | 94.58 | 93.98 | 94.58 | 76.62 | 75.32 | 75.97 |
| DLU_OT2 | 98.18 | 81.82 | 62.75 | 75.47 | 89.09 | 100.00 | 62.75 | 62.75 | 100.00 | 98.18 | 89.09 | - | 99.40 | 100.00 | 77.27 | 75.97 | 76.62 |
| DLU_OT3 | 98.18 | 81.82 | 62.75 | 75.47 | 89.09 | 100.00 | 62.75 | 62.75 | 100.00 | 98.18 | 89.09 | 100.00 | - | 99.40 | 76.62 | 75.32 | 75.97 |
| DLU_OT4 | 98.18 | 81.82 | 62.75 | 75.47 | 89.09 | 100.00 | 62.75 | 62.75 | 100.00 | 98.18 | 89.09 | 100.00 | 100.00 | - | 77.27 | 75.97 | 76.62 |
| DLU_OT5 | 62.75 | 60.78 | 95.92 | 64.00 | 62.75 | 62.75 | 100.00 | 95.92 | 62.75 | 60.78 | 62.75 | 62.75 | 62.75 | 62.75 | - | 96.62 | 97.30 |
| DLU_OT6 | 60.78 | 58.82 | 97.96 | 62.00 | 60.78 | 60.78 | 93.88 | 97.96 | 60.78 | 58.82 | 60.78 | 60.78 | 60.78 | 60.78 | 93.88 | - | 99.32 |
| DLU_OT7 | 62.75 | 60.78 | 100.00 | 64.00 | 62.75 | 62.75 | 95.92 | 100.00 | 62.75 | 60.78 | 62.75 | 62.75 | 62.75 | 62.75 | 95.92 | 97.96 | - |

Note: The lower left corner is the alignment result of the amino acid sequence, and the upper right corner is the alignment result of the nucleotide sequence.
