# Supplementary material for: Molecular epidemiological study of Scrub Typhus in residence, farm and forest habitats from Yunnan Province, China
Source: PLoS One. 2024 Apr 16;19(4):e0301841. doi: 10.1371/journal.pone.0301841 (PMC11020965; doi:10.1371/journal.pone.0301841)
Supplement: S1 File — (DOCX) [file pone.0301841.s003.docx]

>OP925099.1 Uncultured Orientia sp. clone DLU_OT1 56-kDa type-specific antigen gene, partial cds

GCAGAGCTAGGTGTTATGTACCTTACAAATATAACTGCTCAGGTTGAAGAAGGTAAAGTTAAGGCAGATTCTGGAGGTAAGACAAAGGCAGATTCTGGAGGTGGGACAGATGTTCCTATACGTAAGCGGTTTAAACTTACACCTCCTCAGCCTACTATAATGCCTA

>OP925100.1 Uncultured Orientia sp. clone DLU_OT2 56-kDa type-specific antigen gene, partial cds

GCAGAGCTAGGTGTTATGTACCTTAGAAATATAAGTGCTGAGGTTGAATTAGGTAAAGTCAAGGCAGATTCTGGAGGTAAGACAAAGGCAGATTCTGGAGGTGAGACAGATGCTCCTATACGTAAGCGGTTTAAACTTACACCGCCTCAGCCTACTATAATGCCTA

>OP925101.1 Uncultured Orientia sp. clone DLU_OT3 56-kDa type-specific antigen gene, partial cds

GCAGAGCTAGGTGTTATGTACCTTAGAAATATAAGTGCTGAGGTTGAATTAGGTAAAGTCAAGGCAGATTCTGGAGGTAAGACAAAGGCAGATTCTGGAGGTGAGACAGATGCTCCCATACGTAAGCGGTTTAAACTTACACCGCCTCAGCCTACTATAATGCCTA

>OP925102.1 Uncultured Orientia sp. clone DLU_OT4 56-kDa type-specific antigen gene, partial cds

GCAGAGCTAGGTGTTATGTACCTTAGAAATATAAGTGCTGAGGTTGAATTAGGTAAAGTCAAGGCAGATTCTGGAGGTAAGACAAAGGCAGATTCTGGAGGTGAGACAGATGCTCCTATACGTAAGCGGTTTAAACTTACACCGCCTCAGCCTACTATAATGCCTA

>OP925103.1 Uncultured Orientia sp. clone DLU_OT5 56-kDa type-specific antigen gene, partial cds

GCAGAGCTAGGTGTTATGTACCTTGCGAATGTAAAAGCAGAGGTAGAATCAGGTAAAACTGGCTCTGATGCTGATACTAGACTTGGTGCAGATTCTCCTATGCCTCAGCGGTATAAACTTACACCACCTCAGCCTACTATAATGCCTA

>OP925104.1 Uncultured Orientia sp. clone DLU_OT6 56-kDa type-specific antigen gene, partial cds

GCAGAGCTAGGTGTTATGTACCTTGCGAATGTAAAAGCAGAGGTGGAATCAGGTAAAACTGGCCCTGATGCTGATATTAGATCTGGTGCAGATTCTCCTATGCCTCAGCGGTATAAACTTACACCACCTCAGCCTACTATAATGCCTA

>OP925105.1 Uncultured Orientia sp. clone DLU_OT7 56-kDa type-specific antigen gene, partial cds

GCAGAGCTAGGTGTTATGTACCTTGCGAATGTAAAAGCAGAGGTGGAATCAGGTAAAACTGGCTCTGATGCTGATATTAGATCTGGTGCAGATTCTCCTATGCCTCAGCGGTATAAACTTACACCACCTCAGCCTACTATAATGCCTA

>OM914742.1 Orientia tsutsugamushi isolate DALIV8 56-kDa type-specific antigen (tsa56) gene, complete cds

ATGAAAAAAATTATGTTAATTGCTAGTGCAATGTCTGCGTTGTCGTTGCCGTTTTCAGCTAGTGCGATAGAATTGGGGGATGAAGGAGGATTAGAGTGTGGTCCTTACGGTAAAGTTGGAATCGTTGGAGGAATGATTACTAGTGTAGAATCTACTCGCTTGGATCCAGCTGATACTGATGGCAAAAAACATTTGTCATTAACAACCTCGATGCCATTTGGTGGTACATTAGCTGCAGGTATGACAATCGCTTCAGGTTTTAGAGCAGAGATAGGTGTTATGTACCTTAGAAATATAAGTGCTGAGGTTGAATTAGGTAAAGTCAAGGCAGATTCTGGAGGTAAGACAAAGGCAGATTCTGGAGGTGAGACAGATGCTCCTATACGTAAGCGGTTTAAACTTACACCGCCTCAACCTACTATAATGCCTATAAGTATAGCAGATCGTGATTTCGGTGTTGATGTTACTAATATACCTCAAGCTCAAGTACAACCGCCTCAGCAAGCAAATGATCCTCTTGTTCGTGGAGTACGTAGGATTGCTTGGTTAAAAGAGTATGCTGGTATTGACTATATGGTGAAGGATCCTAATAATCCTGGGCGTATGATGGTAAATCCAGTGTTGTTAAATATACCTCAAGGCCCACCTGCTCAAAATCCTAGAGCGGCTATGCAACCTTGTAATATACTTGATCATGATCACTGGAGACATTTGGTAGTTGGTGTCACTGCATTATCAAATGCTAATAAACCTAGCGCTTCTCCTGTCAAAATATTAAGTGAAAAAATTACTCAGATATATAGTGATATAAGGCCATTTGCTGATATAGCTGGTATTAAT

GTTCCTGATACTGGTTTGCCTAATAGTGCGTCTGTCGAACAGATACAGAGTAAAATGCAAGAATTAAGTGATTTATTGGAAGAACTCAGAGATTCTTTTGATGGGTATATTAGTAATGCTTTTGCTGGTCAGATACAGTTGAACTTTGTCATGCCGCCGCAAGCACAGCAGCAGCAGGGGCAAGGGCAGCAACAGCAAGCTCAAGCTACAGCGCAAGAAGCAGTAGCAGCAGCAGCTGTTAGGCTTTTAAATGGTAATGATCAGATTGCGCAGTTATATAAAGATCTTGTTAAATTGCAGCGTCATGCAGGAATTAGGAAAGCCATGGAAAAATTAGCTGCCCAACAAGAAGAAGATTCAAAGAATCAAGGTGAAGGTAGCTGTAAGGTAGATTCTTCTAAAGAAGGAAAAAGCAAAGAGACAGAGTTTGATCTGAGTATGATTGTCGGTCAAGTTAAACTCTATGCTGACTTAATGACAACTGAATCATTCTCAATATATGCTGGTCTTGGTGCAGGGTTAGCTTATACTTATGGAAAAATAGATGATAAGGATATTGGGCATACAGGTATGGTTGCATCAGGAGCACTTGGTGTAGCAATTAATGCTGCTGAGGGTGTGTATGTGGACATAGAAGGTGGTTATATGTACTCATTCAGTAAAATAGAAGATAAGTACTCGGTAAATGCGCTTATTGCAAATGTAGGTGTGCGCTATAACTTCTAG
